# Fluorine-18 fluoro-2-deoxy-D-glucose positron emission tomography/computed tomography scan contributes to the diagnosis and management of brucellar spondylodiskitis

**DOI:** 10.1186/1471-2334-13-73

**Published:** 2013-02-07

**Authors:** Savvas Ioannou, Sofia Chatziioannou, Spiros G Pneumaticos, Alexandra Zormpala, Nikolaos V Sipsas

**Affiliations:** 1 Pathophysiology Department, Medical School, National and Kapodistrian University of Athens, 75, Mikras Asias, 115 27, Athens, Greece; 2 Department of Radiology, Center for Clinical Research, Nuclear medicine and PET/CT Section, Foundation for Biomedical Research of the Academy of Athens, Athens, Greece; 3 Department of Orthopaedics, KAT General Hospital, and Medical School, National and Kapodistrian University of Athens, Athens, Greece; 4 Radiology Department, Laikon General Hospital, and Medical School, National and Kapodistrian University of Athens, Athens, Greece

**Keywords:** Brucella, Spondylodiskitis, Positron emission tomography, Standardized uptake value

## Abstract

**Background:**

Limited data suggest that fluorine-18 fluoro-2-deoxy-D-glucose (F-18 FDG) positron emission tomography combined with computed tomography (PET/CT) scan may be useful for diagnosing infections of the spine. Brucellar spondylodiskitis might be devastating and current imaging techniques lack sensitivity and specificity. The aim of this prospective study was to determine the role of F-18 FDG PET/CT scan in the diagnosis of brucellar spondylodiskitis and in monitoring the efficacy of its treatment.

**Methods:**

Ten consecutive patients with brucellar spondylitis were prospectively evaluated with PET/CT. Baseline evaluation included also magnetic resonance imaging (MRI) of the affected spine, indices of inflammation, the slide agglutination test (SAT), and the standard hematology and biochemistry. All cases were treated with suitable antibiotics until resolution or significant improvement of clinical and radiological (MRI) findings. Upon completion of treatment, they were re-evaluated with follow-up PET/CT scan. The maximum standardized uptake values (SUV) were measured and compared with SAT.

**Results:**

In all patients there was an increased F-18 FDG activity in the infected spine region detected by the initial MRI. F-18 FDG PET/CT provided additional information, compared to MRI, in 4 (40%) patients. More specifically it revealed additional spine lesions (in 3 patients), lymphadenitis, arthritis, organomegaly, as well as new paravertebral soft tissue involvement and epidural masses. This additional information had an impact on the duration of treatment in these patients. At the end of treatment all patients had a complete clinical response; 5 patients had positive serology, 6 patients had residual MRI findings, while 9 had a positive PET/CT but with significantly decreased FDG uptake compared to baseline (median 2.6, range 1.4 – 4.4 vs. median 5.5, range 2.8 – 9.4, p = 0.005). During the follow up period (median 12.5 months) no relapses have been observed. No significant association was observed between the SUV and SAT.

**Conclusions:**

Our study suggests that in patients with brucellar spondylodiskitis F-18 FDG PET/CT scan can provide additional information on the spread of the infection, compared to MRI. Successful treatment is associated with a significant decrease in SUVmax values; thus, PET/CT scan may be a complementary method for determining the efficacy of treatment.

## Background

Brucellosis is a common zoonosis caused by facultative intracellular bacteria of the genus *Brucella*. Osteoarticular involvement is the most common complication of the disease affecting one third of the patients [1]. The most prevalent myoskeletal manifestation is spondylodiskitis, an infection of the intervertebral disk and the adjacent vertebrae, with or without associated epidural or psoas abscesses. Brucellar spondylitis might be a devastating disease, since quite often, it is associated with neurological and vascular complications, requires spinal surgery, and results in permanent functional sequelae [2].

Although it was described back in 1932 [3], there are still controversies regarding its management, such as the best diagnostic strategy, the optimal antibiotic regimen, the duration of treatment, and the criteria to consider the disease as cured [4], since high relapse rates are a major issue. Early and accurate diagnosis is important for reducing permanent spinal deformity or neurological defects. Multiplanar capability and superior tissue contrast make magnetic resonance imaging (MRI) the modality of first choice in the evaluation and follow-up of brucellar spondylodiskitis [5,6]. However, the usefulness of MRI only becomes apparent once a physician suspects the existence of a target lesion. Moreover, residual MRI findings at the end of treatment limit the use of MRI as a criterion to consider the infection as cured.

During the last decade, the combination of fluorine-18 fluoro-2-deoxy-D-glucose (F-18 FDG) positron emission tomography and computed tomography (PET/CT) scan has emerged as a significant molecular imaging technique in clinical oncology and cancer research. Several studies suggested that PET/CT might be a useful modality in patients with infectious spondylitis [4,7-9]. In the current study, our aims were to define whether PET/CT provides additional information, compared to traditional imaging modalities, in the diagnosis of brucellar spondylitis, and to determine whether it can guide physicians to make decisions on the duration of treatment.

## Methods

### Patients and initial evaluation

Ten consecutive patients, who were diagnosed with brucellar spondylitis at the Laikon General Hospital, in Athens were enrolled in this study. The demographic variables recorded at presentation were age, sex, occupation, type of exposure to *Brucella*, associated illnesses, clinical presentation, and diagnostic delay. The baseline haematology studies included total and differential white blood cell counts, haemoglobin levels, erythrocyte sedimentation rate (ESR), C-reactive protein (CRP), the slide agglutination test (SAT) and biochemistry profile. All patients underwent contrast-enhanced MRI of the suspected spine region and were subsequently (within 7 days) referred for a PET/CT scan examination, in order to examine the distribution of active inflammatory lesions and to search for other possible spine lesions not detected earlier by MRI imaging. The study was approved by the Ethics Committee of Athens Laikon General Hospital. All patients gave written informed consent and consented to their individual data being published in this article.

### Diagnosis

The diagnosis of brucellosis was established by the clinical findings compatible with brucellosis, positive SAT (1:160 or higher), and/or by isolating *Brucella* species from blood or bone marrow. Enzyme-linked immunosorbent assay (ELISA) for *Brucella*-specific IgM and IgG serum antibodies, as well as Coombs test were used as additional diagnostic tools where available.

### Imaging studies

Clinical diagnosis of spondylitis was initially established by MRI. The main MR criteria for suspected spondylodiskitis were hypointensity of disc and vertebral bodies on T1-weighted sequences, hyperintensity on T2-weighted sequences and contrast enhancement of the disc, adjacent vertebral bodies and involved paraspinal tissues [10]. All MR images were evaluated both independently and in consensus by 2 experienced radiologists specializing in MRI of the musculoskeletal system, who were blinded to the patients’ clinical history, laboratory, and pathological results.

### F-18 FDG PET/CT protocol and interpretation

All patients fasted for at least 6 hours before the PET/CT study. F-18 FDG was injected intravenously (370 – 555 MBq) and scanning began 60 min later. The serum glucose levels were less than 160 mg/dl before injection. No intravenous contrast was administered. Patients were encouraged to void before imaging and scanned with a combined FDG PET/CT scanner (Siemens Biograph 6 high resolution PET/CT). Patients were imaged in the supine position, with their arms placed above their heads when possible. The acquisition time was 4 min per bed position. CT scans began at the orbitomeatal line and progressed to the upper thigh. PET followed immediately over the same body region. The CT data were used for attenuation correction and images were reconstructed using a standard ordered-subset expectation maximization (OSEM) algorithm. All PET/CT studies were reviewed both independently and in consensus by 2 experienced nuclear medicine physicians, who were also blinded to the clinical, pathological, and imaging results. Positive finding was considered any increased uptake in the vertebral body(ies), disc space(s) and/or surrounding soft tissues compared to the uptake in uninvolved vertebral bodies, disc spaces or soft tissues respectively. The SUVmax of the abnormal areas was recorded.

### Treatment and follow-up period

All patients were treated and followed up according to standard protocols used in our department [11], for at least 6 months. A complete clinical, serology and imaging (MRI) work-up was performed to assess the need for further treatment. Each patient was treated until resolution or significant improvement of clinical and radiological findings. Upon completion of treatment, all patients were re-evaluated with follow-up MRI and within 7 days with PET/CT scanning. Complete remission, partial remission, treatment failure, and relapse were defined as described elsewhere [12].

### Statistical analysis

The SUVmax was calculated at the baseline and follow up PET/CT scan and the difference between the two was analyzed using a Wilcoxon paired test. The difference in median SUVmax in patients with resolution of MRI findings at the end of treatment compared to those with residual findings was determined using a Mann–Whitney *U* test. In addition, Pearson correlation coefficient was used to compare the SUVmax and SAT. A *p* value of less than 0.05 was considered statistically significant.

## Results

### Baseline characteristics

During the study period 10 patients (7 were males) with brucellar spondylitis were enrolled. The median age was 58.5 years (range 37 – 87 years) (Table 1). Two patients had an occupational exposure and all reported ingestion of unpasteurized dairy products. At presentation, all patients experienced fever and pain of the affected spinal region. ESR ranged from 7 to 120 mm/h (median, 67 mm/h) and CRP from 3.08 to 86.8 mg/L (median, 36.8 mg/L). Anaemia was found in 3 (30%) patients and leukocytosis in 4 (40%). Abnormal liver function tests attributed to brucellosis were found in 2 (20%) patients.

**Table 1:** Demographics, baseline laboratory, clinical and imaging findings among the 10 study patients with brucellar spondylitis

**Pt**	**Sex**	**Age**	**ESR (mm/hr)**	**CRP (mg/L)**	**SAT titers**	**WBC (K/μl)**	**Clinical**	**Blood cultures**	**MRI findings (affected vertebrae)**	**F-18 FDG PET/CT findings (affected vertebrae)**	**F-18 FDG PET/CT SUVmax**
1	M	59	106	63.3	1/10240	5.58	Back pain, fever	*Brucella* sp.	Spondylodiscitis L2 – L3, L5 – S1, paravertebral masses	Spondylodiscitis T12 – L3, L5 – S1, paravertebral masses, mediastinal lymph node involvement	5.5 2.8
2	M	61	14	10.1	1/10240	8.10	Back pain, fever	(−)	Spondylodiscitis T7 – T8, paravertebral mass	Spondylodiscitis T7 – T8, L4 – L5, paravertebral and epidural masses	7.1 4.4
3	F	83	7	3.08	1/20480	5.93	Back pain, fever	(−)	Spondylodiscitis L2 – L3	Spondylodiscitis L2 – L3,	6.7
4	M	51	74	51.5	1/1280	13.30	Pack pain, fever	(−)	Spondylodiscitis L4 – L5, paravertebral mass	Spondylodiscitis L4 – L5, T5 – T6, paravertebral masses, arthritis Lt acromioclavicular joint	5.4 5.8
5	M	53	80	38.6	< 1/160	9.30	Back pain, fever	(−)	Spondylodiscitis L4 – L5	Spondylodiscitis L4 – L5	6.9
6	F	71	120	86.8	1/2560	6.50	Back pain, fever	*Brucella* sp.	Spondylodiscitis L4 – L5, paravertebral mass	Spondylodiscitis L4 – L5, paravertebral mass	9.4
7	F	37	60	24	< 1/160	14.70	Back pain, fever	(−)	Spondylodiscitis T6 – T7	Spondylodiscitis T6 – T7, hepatosplenomegaly	3.0
8	M	46	80	18.5	< 1/160	9.63	Back pain, fever	(−)	Spondylodiscitis L2 – L3, epidural mass	Spondylodiscitis L2 – L3, epidural mass	5.5
9	M	58	40	60	< 1/160	12.50	Back pain, fever	(−)	Spondylodiscitis L3 – L4	Spondylodiscitis L3 – L4	6.4
10	M	87	80	67	1/2560	11.40	Back pain, fever	(−)	Spondylodiscitis L1 – L2, L4 – L5, paravertebral mass	Spondylodiscitis L1 – L2, L4 – L5, paravertebral mass	5.2 6.0

NOTES. All patients provided written consent to publish these details. Pt: patient number, ESR: erythrocyte sedimentation rate (normal value, 40 mm/hour), CRP: C-reactive protein (normal values < 5 mg/L), WBC: white blood count, SAT: slide agglutination test (normal values < 1/160), MRI: magnetic resonance imaging, F-18 FDG PET/CT: fluorine-18 fluoro-2-deoxy-D-glucose positron emission tomography combined with computed tomography scan, SUVmax: maximum standard uptake value.

SAT of initial samples was positive in 6 (60%) of the 10 patients. In the rest with negative SAT, a diagnostic CT-guided biopsy of the affected spinal region was performed. Biopsy results showed diffuse nonspecific inflammation with noncaseating granulomatous fibrous deposits. Furthermore, in all the biopsy samples additional microbiologic and histologic examinations were performed which revealed the presence of *Brucella sp*. ELISA was performed in 4 patients and revealed high antibody levels in 2. Blood cultures were positive in 2 patients (20%) and all grew *Brucella sp.*; susceptibility tests were not performed.

### Imaging findings

All patients underwent an MRI scan at presentation, at the vertebral level suspected to be infected by *Brucella*, according to history and clinical findings. In all cases, MRI had led to the strong suspicion of spondylodiskitis. The vertebral level involved was lumbar or lumbo-sacral in 8 (80%) cases and thoracic in 2 (20%) cases. Five patients had paravertebral soft tissue involvement, while one had epidural mass (Table 1).

**Figure 1:**
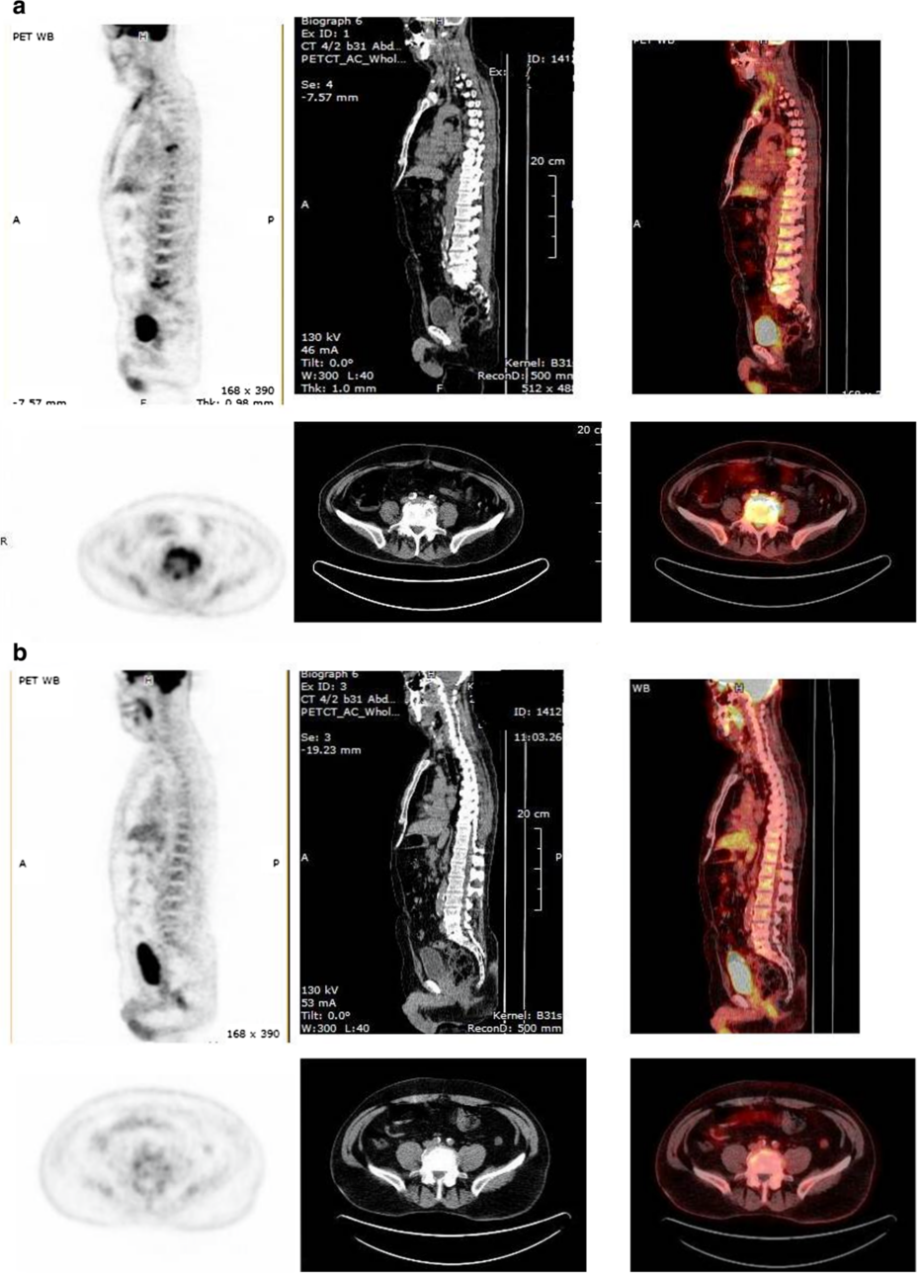
**A. Sagittal (top) and coronal images of baseline PET/CT scan.** Detailed legend: These images of patient no 4 show abnormal hypermetabolism at the left acromioclavicular joint, and at T6 and L5 vertebrae, along with paravertebral soft tissue involvement. Initial MRI evaluation of the lumbar spine of the same patient revealed only the lesion at the area of L4 – L5 vertebrae with paravertebral mass. **B.** Follow-up PET/CT scan after treatment. Detailed legend: These images of patient no 4 post-treatment demonstrate decreased FDG uptake at T6 and L5 vertebrae, and no uptake at the paravertebral tissues.

Baseline PET/CT scan was performed subsequently in all patients. In all 10 cases there was an increased F-18 FDG activity in the infected spine region, as diagnosed by MRI (Table 1). In 4 patients the initial PET/CT scan provided additional information compared to MRI, such as lesions at different spine levels (3 patients), mediastinal lymphadenitis, arthritis, organomegaly, as well as new paravertebral soft tissue involvement and epidural masses. More specifically, additional spine lesions have been detected: in patient 1 (T12 – L1, L1 – L2); in patient 2 (L4 – L5); and in patient 4 (T5 – T6) (Table 1) (Figure 1a). These patients underwent additional MRI scans of the affected new spine region which confirmed the presence of spondylodiskitis. The median SUVmax in the 14 affected vertebrae was 5.5 (range 2.8 – 9.4). An SUVmax value ≥ 3.0 was observed in 13 of the 14 affected vertebrae.

### Follow-up and outcome

The duration of treatment was based on clinical criteria and imaging (MRI) re-evaluation. Only two patients (Table 2, patients 5 and 9) had complete remission at 6 months and discontinued treatment. Six patients (Table 2, patients 1, 2, 4, 7, 8, 10), including those with additional PET/CT scan findings, had partial remission at 6 months, and received antibiotic treatment for 12 months. More specifically, they continued to experience pain of lesser severity at the affected spinal areas with residual imaging findings. One patient (Table 2, patient 3) discontinued treatment at approximately 9 months due to immune thrombocytopenia which was attributed to the antibiotics, and patient 6 discontinued treatment at 11 months, when complete remission was noted. At the end of treatment all patients had complete remission of the disease with no residual paravertebral or epidural masses. After treatment discontinuation all patients were followed up with regular visits at the outpatient clinic for a median period of 12.5 months (range 8 – 27). No recurrences were observed during this period; therefore, we consider that treatment was successful in all of our study patients.

**Table 2:** Laboratory, clinical and imaging findings of the 10 study patients with brucellar spondylitis at the end of treatment

**Pt**	**Duration of treatment (months)**	**Findings at the end of treatment**								**Duration of follow-up after treatment discontinuation (months)**
		**ESR mm/hr**	**CRP mg/L**	**SAT titers**	**WBC K/μl**	**Clinical**	**MRI residual findings**	**F-18 FDG PET/CT findings**	**F-18 FDG PET/CT SUVmax**	
1	12	40	5.00	1/320	4.70	(−)	L5 – S1	L2 – L3, L5 – S1	3.2 2.6	27
2	12	10	5.00	1/680	6.50	(−)	T7 – T8	T7 – T8, L4 – L5	3.3 2.0	13
3	9	5	3.08	1/ 10240	4.43	(−)	L2 – L3	L3	2.9	21
4	12	35	10.00	1/320	6.65	(−)	L4 – L5	L5 T6	2.6 2.9	10
5	6	15	2.97	< 1/160	7.24	(−)	(−)	(−)	0	9
6	11	15	2.97	1/320	5.15	Back pain on effort	L4 – L5	L4 – L5	2.7	8
7	12	17	3.08	< 1/160	7.85	Pain on weather changes	(−)	T7	2.0	17
8	12	20	4.10	< 1/160	8.90	(−)	(−)	L3	2.0	11
9	6	14	5.00	< 1/160	7.70	(−)	(−)	L3	3.0	21
10	12	23	5.00	< 1/160	4.80	Back pain on effort	L4 – L5	L1 – L2, L4 – L5	2.9 4.4	12

NOTES. Pt: patient number, ESR: erythrocyte sedimentation rate (normal value, 40 mm/hour), CRP: C-reactive protein (normal values < 5 mg/L), WBC: white blood count, SAT: slide agglutination test (normal values < 1/160), MRI: magnetic resonance imaging, F-18 FDG PET/CT: fluorine-18 fluoro-2-deoxy-D-glucose positron emission tomography combined with computed tomography scan, SUVmax: maximum standard uptake value.

At the end of treatment all patients had negative indices of inflammation with normal full blood count and liver function tests. In all 10 patients a follow-up PET/CT scan was performed after treatment completion (Table 2). Only one patient (Table 2, patient 5) had negative PET/CT scan findings; in the rest 9 patients the PET/CT scan findings were diagnosed as positive. However, a visual decrease in the F-18 FDG uptake compared to the uninvolved tissues, with median SUVmax 2.6 (range 1.4 – 4.4) was observed in the 14 infected vertebrae (Figure 1b). An SUVmax value > 3.0 was observed in only 3 of the 14 affected vertebrae. Complete resolution of MRI findings occurred in 4 patients (Table 2, patients 5, 7, 8, 9) at the time of the last follow-up. Three of these patients had positive PET/CT scan findings; SUVmax was significantly lower in patients with resolution of MRI findings, compared to patients with residual MRI findings (median SUVmax 2.0 vs. 3.05, p = 0.05). No significant association was observed between the SUVmax and SAT values (p > 0.05).

## Discussion

In the present, it was demonstrated that PET/CT scan may provide useful information in the management of brucellar spondylodiskitis. All patients enrolled had positive PET/CT scan findings at the time of diagnosis, with high uptake of FDG within the infected tissue, as SUVmax values were as high as 9.4, and 13 of the 14 infected vertebrae had SUVmax > 3.0. More importantly, in 4 (40%) of our patients, PET/CT scan provided additional information on the spread of the infection, beyond the initial lesion detected by MRI. This is particularly important as MRI of the whole spine is not logistically feasible and other noncontiguous foci of spinal infection can be missed, exposing patients to the risk of devastating neurological complications. Moreover, brucellosis is a systematic disease affecting many organs and systems; therefore, PET/CT scan is suitable to define the real extend of the disease, beyond the affected spine.

Our data are in accordance with those of other studies that demonstrated high sensitivity and specificity of PET/CT scan in detecting infectious processes of the spine [13,14]. A recent review article [15] highlights the clinical role of this technique in diagnosing spinal infections, with sensitivities ranging from 94% to 100% and specificities ranging from 87% to 100%. Moreover, in a recent retrospective study [16] PET/CT had a strong impact on the clinical management of 52% of patients with infectious spondylitis.

Usefulness of PET/CT in monitoring the efficacy of treatment was less clear; at the end of treatment only 1 patient had a negative PET/CT while 4 patients had a negative MRI of the affected spine. Successful treatment resulted+ in a significant decrease in SUVmax values, while patients with no residual MRI findings had significantly lower SUVmax compared to those with residual MRI findings. As expected, after successful treatment virtually all patients had negative indices of inflammation.

MRI is the imaging modality of choice for the diagnosis of brucellar spondylodiskitis as it has high sensitivity and specificity, but it has technical limitations: it is sensitive to motion degradation, so that patients with movement disorders may not be suitable candidates; certain metallic implants are contraindications for this modality; it cannot always help distinguish spondylitis from severe degenerative arthritis. Moreover, physicians order MRI scans only of the spinal region with the suspected target lesion. However, in published series of patients with brucellar spondylodiskitis a substantial percentage of up to 9% [2,17] had noncontiguous multifocal spinal involvement, which can be missed if only one spinal region is assessed. Therefore, in every case of suspected brucellar spondylitis the whole spine should be evaluated with MRI, but this is logistically difficult in the everyday practice.

Several clinical studies [7,13,18,19] have reported the effectiveness of PET in imaging musculoskeletal infections. PET allows rapid imaging within 1 h after the FDG-injection [18]. Additionally, FDG-PET shows a high spatial resolution. This can be helpful in assessing the paravertebral abscesses in spondylodiskitis. On the other hand, FDG-PET/CT is not always possible to distinguish between spondylodiskitis and degenerative changes in the vertebral endplates. Stumpe et al., found that none of the patients in their study with degenerative disc disease had increased uptake on PET imaging [19]. Conversely, Rosen et al. found increased disc activity in those with degenerative changes [20]. In another study, Schmitz et al. found that all the cases in their series that had histopathological confirmation of infection had positive FDG PET imaging [18].

**Figure 2:**
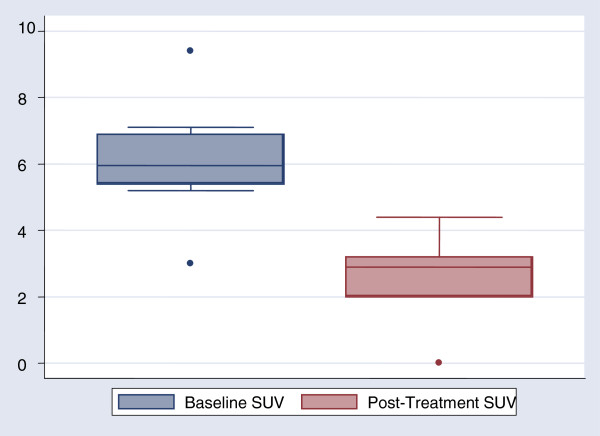
SUVmax at baseline PET/CT scans, before treatment initiation vs. post-treatment PET/CT scan, showing a significant decrease (p = 0.005).

The optimal duration of treatment of brucellar spondylodiskitis remains a controversial issue. Serology and imaging are not always helpful, as residual MRI findings are present long after successful treatment [21]. We consider that all our cases were treated successfully, because no relapses have been observed during the follow up. Nevertheless, at the end of treatment 5 of the 10 patients had a high SAT titer, while only 4 had a negative MRI of the affected spine. Successful treatment resulted in a significant decrease in median SUVmax values from 5.5 to 2.6 (Figure 2), while patients with no residual MRI findings had significantly lower SUVmax compared to those with residual MRI findings. The reason of FDG uptake, even in patients with no residual MRI findings, and despite successful treatment is not clear. One possible explanation is that even successful treatment does not eliminate the infection but it turns it to a latent infection of the spine. Actually, recent studies have shown that long after discontinuation of treatment serology and serum PCR for *Brucella* remain positive despite the lack of clinical symptoms [22,23].

Our study did not detect an SUVmax cut off value for successfully treated patients. However, an SUVmax value > 3.0 was observed in only 3 of the 14 affected vertebrae after successful treatment compared to 13 before treatment initiation. These findings suggest that SUVmax < 3.0 might be indirect evidence of successful treatment, along with negative indices of inflammation, resolution or improvement of MRI findings, and clinical improvement.

This study is limited by the small number of patients and the lack of control group, because brucellar spondylodiskitis is a rather rare disease. However, we have shown that in patients with brucellar spondylodiskitis PET/CT scan provided additional information on the spread of the infection, beyond the initial lesion detected by MRI. Moreover, our study showed that successful treatment is associated with a significant decrease in SUVmax values; thus, PET/CT scan may be a complementary method to MRI for determining the efficacy of treatment. More studies are clearly required to identify the role of PET/CT imaging in the management of patients with brucellar spondylitis.

## Conclusions

To our knowledge, this is the only study to date to evaluate the role of F-18 FDG PET/CT scan in the diagnosis and efficacy of treatment of patients with brucellar spondylitis. Brucellar spondylitis might be a devastating disease, since quite often, it is associated with neurological and vascular complications, requires spinal surgery, and results in permanent functional sequelae. MRI remains the imaging procedure of choice for the evaluation and assessment of therapy of this complication. However, two interesting findings emerge from our study: firstly, F-18 FDG PET/CT scan can provide additional information on the spread of brucellar spondylitis, compared to MRI; secondly, successful treatment is associated with a significant decrease in SUVmax values. Therefore, PET/CT scan may be useful in assessing the efficacy of treatment and the need for further antimicrobial chemotherapy. Further research is necessary to better understand the role and impact of PET/CT imaging in the management of patients with brucellar spondylitis.

## Abbreviations

F-18 FDG: Fluorine-18 fluoro-2-deoxy-D-glucose; PET/CT: Positron emission tomography combined with computed tomography; MRI: Magnetic resonance imaging; SAT: Slide agglutination test; SUV: Standardized uptake value; ESR: Erythrocyte sedimentation rate; CRP: C-reactive protein; ELISA: Enzyme-linked immunosorbent assay.

## Competing interests

The authors declare that they have no competing interests, with respect to this article.

## Authors’ contributions

Each author has participated sufficiently in the work to take public responsibility for appropriate portions of the content. NVS and SGP conceived of the study, and participated in its design and coordination and helped to draft the manuscript. SI participated in the design of the study and performed the statistical analysis. SC and AZ performed the imaging studies. All authors read and approved the final manuscript.

## Pre-publication history

The pre-publication history for this paper can be accessed here:

http://www.biomedcentral.com/1471-2334/13/73/prepub
